# Closing the Gap Between Emerging Initiatives and Integrated Strategies to Strengthen Science Diplomacy in Latin America

**DOI:** 10.3389/frma.2021.664880

**Published:** 2021-04-12

**Authors:** Sandra López-Vergès, Lorena Macías-Navarro, Alma Cristal Hernández-Mondragón, Eugenia Corrales-Aguilar, Marga Gual Soler, Melania Guerra

**Affiliations:** ^1^Department of Research in Virology and Biotechnology, Gorgas Memorial Institute of Health Studies, Panama City, Panama; ^2^México Actúa, México City, Mexico; ^3^Program on Science, Technology and Society, Center for Research and Advanced Studies of the National Polytechnic Institute, México City, Mexico; ^4^Virology-CIET (Research Center for Tropical Diseases), Microbiology, University of Costa Rica, San José, Costa Rica; ^5^SciDipGLOBAL, Mallorca, Spain; ^6^Department of Environment and Development, University for Peace, Ciudad Colón, Costa Rica

**Keywords:** Latin America, science diplomacy, science-technology-innovation policy, science-based, evidence-based decisions, sustainable development goals, agenda 2030 for development

## Abstract

Science diplomacy is a fast-growing field of research, policy, and practice dedicated to understanding and reinforcing the connections between science and international affairs to tackle national, regional, and global issues. By aligning science and diplomacy, countries can attract talent, strengthen their national research ecosystems, provide avenues for participation of scientists in policy, and coordinate integrated solutions to challenges with technical dimensions. While Latin America has a long tradition of bilateral and regional cooperation, science still plays a marginal role in foreign policy, as has become evidenced by the response to the COVID-19 pandemic. With few exceptions, Latin American nations have a relatively immature science, technology, and innovation ecosystem, compounded by low public and private investments in research, coexisting with profound socio-economic inequalities, and large vulnerable populations. Such challenging conditions have created barriers to a fluid relationship between science and diplomacy, fundamentally characterized by inefficient communication between scientists and policymakers, weak collaboration channels, and duplicated roles, which altogether perpetuate siloed mentalities and a lack of trust between the two communities. Over the last decade, a first influential wave of Latin American scientists, diplomats, and other professionals, including five of the co-authors, have undertaken science diplomacy training provided by specialized organizations. Through these experiences, we recognized the need to elevate awareness and build capacities in science diplomacy in our respective countries and overall, across Latin America. Here, we describe emerging efforts and mechanisms to bridge the gap between scientists and policymakers at the national and regional level. Furthermore, we offer recommendations to amplify the impact of those pioneering initiatives toward consolidating a robust science diplomacy practice across the region. The national experiences described from Costa Rica, Mexico, and Panama can serve as a roadmap for other Latin American nations in the early process of developing a science diplomacy strategy, so they can also align themselves to a collective pathway. Most critically, we propose a way forward so that Latin America can leapfrog beyond disjointed training of individuals into integrated institutional strategies that can harness the tools of science diplomacy to enhance science-informed multilateral cooperation and enable more effective science-informed policymaking.

## Introduction

Although science and diplomacy have been intertwined throughout modern history, the term was only coined in the early twenty-first century (Lord and Turekian, 2007). Science diplomacy is considered an umbrella term describing a wide range of policies and practices at the intersection of science and international affairs to advance national, regional, and global interests (Royal Society, 2010). Among the manifold objectives of nations engaging in science diplomacy are attracting scientific talent from abroad, strengthening national research, and innovation systems, promoting a “country brand” on the global stage, providing avenues for the participation of scientists in the formulation of public policies and coordinating integrated solutions to regional problems.

While Latin America has a long tradition of bilateral and regional cooperation on many topics and issues, science still plays a marginal role in foreign policy and regional integration. With few exceptions, Latin American nations have a relatively immature science, technology, and innovation (STI) ecosystem (IDB, 2010), compounded by some of the lowest public and private investments in STI (Tomilova and Dashi, 2019), all coexisting with profound socio-economic inequalities and large vulnerable populations (UNDP, 2019).

Such challenging conditions, along with deep ideological fractures, have created complex barriers to connecting scientific and diplomatic structures (Gual Soler, 2014) to collectively respond to transboundary emergencies, such as climate change or the ongoing COVID-19 pandemic^1^. Furthermore, most of the intellectual foundations and practical applications of science diplomacy have emerged in the Global North, resulting in a lack of resources in Spanish and contextualized examples to the Latin American region.

Recognizing the immense potential and urgency to develop endogenous science diplomacy capacities in the region, a generation of young Latin American leaders, including five of the co-authors, have sought training at various science diplomacy programs and workshops around the world. Among the most recognized initiatives are those led by the American Association for the Advancement of Science (AAAS, based in Washington, DC), The World Academy of Sciences (TWAS, in Trieste), the European S4D4C project, the SciTech DiploHub (in Barcelona), the International Network for Government Science Advice (INGSA, based in Auckland), and the Innovation and Science Diplomacy School (InnSciDSP in São Paulo) (Mauduit and Gual Soler, 2020). Latin American participants represent a minority in these trainings: at the TWAS courses offered between 2015 and 2020, only 15% of partakers came from Latin American or Caribbean countries (Gual Soler et al., 2020). The InnSciDSP program recruits 50% of its participants from Brazil and the other 50% from abroad, a step in the right direction toward building relevant capacity in the region (Anunciato et al., 2020). Yet despite growing interest, participation in these trainings is still mainly driven by individual or institutional motivation, disconnected from cohesive, larger-scale national strategies.

Five of the co-authors of this article, originally from Costa Rica (MG, ECA), Mexico (AH, LM), and Panama (SLV) met at the 2017 edition of the Science Diplomacy and Leadership Workshop of the AAAS Center for Science Diplomacy, organized by MGS, then Senior Project Director at the AAAS Center for Science Diplomacy^2^. The program, a weeklong immersion in the science diplomacy ecosystem of Washington DC, included expert lectures, institutional visits to U.S. agencies like the Department of State and NASA, foreign embassies, and international organizations. It also trains participants in negotiation skills, cross-cultural communication, and policy understanding. As experts in a variety of scientific disciplines, including chemistry, oceanography, nutrition, virology, and cell biology, we were motivated to bring our scientific expertise to bear in policymaking for the sustainable development of our region.

Upon our return from the program, we engaged with key science and policy stakeholders in our respective countries to help build and strengthen their science diplomacy ecosystems. Here, we present the journey of our countries—Costa Rica, Mexico, and Panama—in science diplomacy and document some of the advances and transformations we have contributed to and their impacts to date. Based on our experiences, we conclude by proposing suggestions for scaling capacities in science diplomacy across Latin America and provide recommendations to better integrate these pioneering initiatives into the sustainable development strategies of individual countries and the region as a whole.

## Three National Journeys in Science Diplomacy: Costa Rica, Mexico, and Panama

### Costa Rica

In Costa Rica, close collaborations between the scientific and diplomatic spheres have been ongoing for decades. As one of the first countries to demilitarize, Costa Rica prioritized resources for education, culture, healthcare, and environmental conservation, championing diplomacy, and peaceful engagement over hard power. Back in 1997 Costa Rica (together with Malaysia) proposed a model Nuclear Weapons Convention, in 2013 chaired the first Open-Ended Working Group on nuclear disarmament and eventually marshalled the Treaty on the Prohibition of Nuclear Weapons (TPNW) negotiation conference, which was adopted in 2017 (Umaña, 2018). Not surprisingly a Costa Rican diplomat, Christiana Figueres, led the negotiations efforts at the UN Framework Convention on Climate Change (UNFCCC) toward the Paris Agreement (Figueres, 2020).

Nevertheless, all these efforts only began to be labeled and recognized as “science diplomacy” after 2014, with the appointment of a scientist (Dr. Román Macaya) as Costa Rican Ambassador to the United States. In this role (a first for a scientist to be named ambassador), Dr. Macaya implemented an unprecedented agenda of scientific cooperation between the two countries, resulting in highly impactful collaborations in water, public health, disaster prevention, and remote sensing. In 2015, one such collaborative project linked researchers from the United States Geological Survey (USGS) with the Ministry of Environment and Energy (MINAE), leading to the production of high-resolution, country-scale remote-sensing mapping of underground aquifers (Belcher et al., 2019). This knowledge enables Costa Rica to create evidence-based policies for the use and management of its groundwater resources, which are vital to economically crucial activities such as agriculture and tourism and are especially stressed under a changing climate (Cuadrado-Quesada et al., 2018).

In 2017 another memorandum of understanding (MOU) signed between the University in Costa Rica (UCR), the Costa Rican Social Security (CCSS), and George Mason University, originally designed to produce collaborations on infectious diseases such as zika and dengue, facilitated joint work in the formulation and testing of potential Covid-19 treatments during the 2020 pandemic^3^. This example highlights how international scientific partnerships, when sustained over time, can become advantageous in unexpected ways, most critically during crises.

Other science diplomacy efforts have leveraged strengths in local scientific production to shape diplomatic agendas. For example, research results from the Clodomiro Picado Institute were used as a basis for a 2016 proposal before the World Health Assembly, in pursuit of integrated global action to reduce deaths and disability caused by snake bites. This diplomatic initiative culminated in the 2017 declaration of snakebite poisoning as a priority neglected tropical disease by the World Health Organization (WHO) (Gutiérrez et al., 2017).

In order to formalize these interactions, which so far have been largely *ad-hoc* and serendipitous, in 2019 the Ministry of Foreign Affairs, through its Manuel María de Peralta Foreign Service Institute and the National Academy of Sciences of Costa Rica (ANC), began institutional efforts to link diplomats with researchers through regular convenings. Among the strengths in domestic scientific capacity, that could be leveraged to address relevant regional issues are the prevention of natural disasters through collaboration with the Volcanological and Seismological Observatory (OVSICORI), the conservation of marine biodiversity through the Center for Research in Marine Sciences and Limnology (CIMAR), and biotechnological development through the National Center for Biotechnological Innovations (CENIBiot). At the bilateral level, a recent example of transboundary science-based collaboration between foreign ministries is the joint submission of Costa Rica and Ecuador, requesting to extend their maritime boundaries over the continental shelf between the oceanic islands of Cocos (Costa Rica) and the Galapagos (Ecuador), with the added goal of jointly protecting pelagic species along the migratory passageway, which are threatened by illegal fishing in the area^4^.

More recently, with heightened public awareness about the urgency to integrate science into foreign decision making, prompted by the Covid-19 pandemic, a series of virtual webinars were focused on science diplomacy. Last August, the UNESCO office in San José convened the first ever high-level panel in Costa Rica dedicated to the topic, featuring top representatives from the Ministry of Science and Technology (MICITT), the Ministry of Foreign Affairs, and other public entities. A more informal platform called Halo Sessions, geared toward informing the general public, also organized a webinar on science diplomacy in June 2020. Both of the Costa Rican co-authors (MG and ECA) participated in these events. And in early 2021, co-author MGS was invited by the Ministry of Foreign Affairs to present the results of her UNESCO report on science diplomacy in Latin America and the Caribbean to Costa Rican diplomats and invited scientists.

Slowly, these incremental efforts are permeating into the institutional structures. However, many barriers still remain for early-career scientists to follow in Dr. Macaya's footsteps: just like in most countries STEM graduates are not traditionally exposed to science diplomacy curricula nor are there frequent spaces of interaction with diplomats. On the other hand, the Ministry of Foreign Affairs is currently developing a science diplomacy strategy, which would be formalized into the Process of Economic Diplomacy. Furthermore, they are actively seeking cooperation opportunities with science diplomacy e-training platforms that could provide courses on this topic to their personnel.

### Panama

Panama has a young science, technology, and innovation system, even if science and technical advances have been crucial since its early national development. Historical scientific institutions, such as the Smithsonian Tropical Research Institute (STRI) and the Gorgas Memorial Institute^5^ focused on tropical medicine (Wright, 1970; Adames, 2003), were created at the beginning of the twentieth century to control epidemics during the construction of the Panama Canal. Initially these institutions were administered by the United States, but thanks to close collaboration with local authorities and universities, they have played a central role in advancing Panama's scientific output in public health, biodiversity conservation, and in the training of Panamanian scientists (Sholts et al., 2021). Many new viruses discovered in Panama were first described at the Gorgas, and the results of oceanographic research on underwater acoustics conducted by STRI led the International Maritime Organization (IMO) to modify ship routes and speed limits entering the Panama Canal, with the goal of reducing the risk of humpback whale vessel collisions (Guzman et al., 2020). The BioMuseo, a biodiversity museum, has become a popular tourist attraction with an important role in the education of the general population. However, it was not until the early twenty-first century that these science diplomacy initiatives began to be recognized as such.

In 2017, the National Secretariat for Science, Technology, and Innovation (SENACYT), the science funding agency responsible for developing and strengthening the country's research and innovation systems, selected two young scientists from Panama (co-author SLV was one of them) to participate in the AAAS Science Diplomacy and Leadership Workshop. During our time in Washington DC, we met with a representative of the Panamanian Embassy and the discussion revealed the lack of communication between the diplomatic and the scientific spheres in Panama. It also manifested that most governmental institutions in Panama did not know much about the small but rapidly growing scientific community in the country, nor the breadth of domestic expertise in different scientific fields. Upon our return to Panama, we began conversations to raise awareness about science diplomacy among different stakeholders and institutions. SENACYT and the Ministry of Foreign Affairs (MOFA) approached the scientific community and international partners, including AAAS, UNESCO, and the Inter-American Institute for Global Change Research (IAI), to organize the first science diplomacy workshop in the country as a side event of the Latin American Open Science Forum (CILAC 2018), with the objective of raising awareness among scientists, diplomats, decision-makers, and journalists about the benefits and potential of science diplomacy to support the science system in Panama as well as the role of science to help meet the UN Sustainable Development Goals. Other Panamanian diplomats and scientists, many belonging to the Panamanian Association of the Advancement of Science (APANAC)^6^ and to the #CienciaEnPanama movement^7^, which seeks to augment science communication and science-informed policy making, continued their international training at the SciTechDiploHub course in Barcelona and the European S4D4C project virtual training, both with the participation of MGS as speaker and/or facilitator.

In 2018 Panama became the first Latin American country to launch an official national strategy for science diplomacy, championed by former Vice President and Foreign Minister Isabel de Saint Malo, in coordination with SENACYT. The strategy establishes that the new diplomatic cadres must be familiar with science, technology, and innovation, and new institutional capacities must be created within Panama's foreign policy structures to align national and international policies with the 2030 Agenda. The action plan included the establishment of a Science Diplomacy Committee to foster dialogue and collaboration between the government and the scientific community. One of the key elements of the strategy was to incentivize the recruitment of STEM professionals to diplomatic careers, so Parliament updated the rules regarding the requirements for entry into the foreign service to open it to graduates from any background (Decreto Ley 60, 2015). The Diplomatic Academy incorporated a module dedicated to science diplomacy, with the participation of several scientists trained in the AAAS, S4D4C, and SciTechDiploHub programs as facilitators. After the launch of the strategy, the institutionalization of science diplomacy is progress, and it is expected to be included in the new Science Law to be approved during 2021. An important takeaway from the Panama experience is that science diplomacy is being used as a model for deploying science advice mechanisms at the domestic level. The MOFA is setting the example for other government institutions on the need to take scientific knowledge into consideration to solve many complex national and regional issues in health, agriculture, environment, and more. Panama is thus an example of how the successful combination of high-level and bottom-up leadership can help position a small country at the forefront of science diplomacy in the region.

### Mexico

Over the past 30 years, Mexico has taken important steps toward strengthening institutional capacities to connect science and policymaking, both through science advice and science diplomacy. In 1989, the Federal Government created the first Science Advisory Council (Consejo Consultivo de Ciencias or CCC)^8^, aimed at providing scientific advice and technical support to the Office of the President. The CCC is composed of distinguished researchers awarded with the National Science and Arts Prize, who participate on an honorary basis and rely on the technical and operational support of an Executive Secretariat to coordinate and communicate with the Federal Government. In 2009, the Center for Research and Advanced Studies of the National Polytechnic Institute of Mexico, supported by several members of the CCC, created a transdisciplinary Ph.D. program to train researchers to analyze the interface between science and technology to address pressing social needs^9^. At this time, the program started to prepare young researchers for opportunities they had not yet envisioned.

As the governance requirements of the science, technology, and innovation ecosystem grew, there was a need to create a dedicated office within the Mexican Government that would link the scientific community with policymakers more effectively. In 2013, the Science, Technology, and Innovation (STI) Office was established within the Office of the Presidency, elevating science advice to the highest political level.

At this point, there was enough momentum within the scientific and policy communities, and opportunities to work between these two worlds started to materialize. Between 2012 and 2018, a new wave of young scientists interested in policy and diplomacy began to emerge, including co-authors AH and LM. We too participated in the 2017 AAAS Science Diplomacy and Leadership Workshop in Washington DC, where we connected with other scientists and diplomats from around the globe and joined a powerful network of collaboration and support. Back in Mexico, we started building bridges between our national research institutions and the international community, by fostering collaboration between the CCC, the President's STI Office, the National Council of Science and Technology (CONACYT), and international organizations such as AAAS^10^, the International Network for Government Science Advice (INGSA), the Foreign Ministries Science and Technology Advice Network (FMSTAN)^11^, and the European Science Diplomacy Cluster.

In 2017, the STI Office, the CCC, and the AAAS Center for Science Diplomacy co-organized the *1st Mexican Congress on Science-Informed Policy: Enhancing the Science-Policy Interface*^12^ with the overarching goal of bringing together the science and policy communities in Mexico and the Americas^13^. The program was intended to provide diverse perspectives on the existing mechanisms and models that different countries were deploying to strengthen the science-policy interface. A concrete outcome of the event was a collaboration between CCC, AAAS, and the Diplomatic Academy of the Ministry of Foreign Affairs (Instituto Matías Romero) to develop an online course on science diplomacy and the opportunities and challenges of the Fourth Industrial Revolution for the members of the Mexican Foreign Service and officials of the Foreign Ministry. The course, developed by LM and MGS, was the first to be introduced at the Diplomatic Academy on this topic, and now has become part of the educational curriculum of Mexican diplomats.

The efforts to strengthen science-policy interfaces in Mexico were not limited to the Executive Branch. In the Federal Legislative Branch, an Office of Scientific and Technological Information for the Congress of the Union (INCYTU) was established and operated by the Scientific and Technological Consulting Forum (FCCyT) between 2016 and 2018. However, as it was external to Congress and financially dependent on the Executive, it had a modest impact and disappeared early 2019. In 2021, a new proposal has been passed to create an internal Office for Science Advice in the Chamber of the Deputies, which will depend on the Congress both financially and structurally. Additionally, the Federal Law of Science and Technology will be updated as a consequence of a Constitutional amendment. The objective of this law must include mechanisms for strengthening the institutional frameworks for the long-term development of science and technology, to consolidate the role of advisory bodies and research organizations such as the CCC, and to recommend the creation of new structures dedicated to science diplomacy and science advice not only in the executive and legislative branches, but in other levels of governance. An example at the sub-national level is a science policy fellowship program hosted by the Government of Mexico City (CDMX), led by AH and inspired in the AAAS Science Technology and Policy Fellowship (STPF), to place Ph.D. scientists in government offices for 1 year to develop knowledge and skills to navigate the science and public policy nexus^14^. The program is supported by AAAS (USA), FECYT (Spain), IIASA (Austria), and the United Kingdom, who helped deliver orientation training to help fellows prepare for their placement and identify and develop their transferable skills. The first cohort of the program—the first of its kind in Latin America—has placed fellows in the Health, Environment, Economic Development, and Mobility Ministries in CDMX and will be expanded to other ministries in the coming years.

## The Way Forward: From Emerging Initiatives to Integrated Strategies

The COVID-19 pandemic response has manifested the divide between science, policy and society in many countries, and Latin America is no exception. Despite the promising advances presented here, our countries are still reactive, rather than anticipatory, to the challenges that require robust scientific input and regional and global cooperation. Governments must adopt this new vision of development and prosperity based on strengthening their science, technology, and innovation systems, and connecting knowledge to policy and society. Much more needs to be done to foster local and international cooperation to strengthen communication channels between scientists, diplomats, and policy makers. We show how the leadership of young pioneers, the co-authors being only few examples, supported by and trained in international programs have been a crucial first step to create a group of champions of science diplomacy in the region.

First, national and regional institutions should take the lead in establishing regular capacity development in science diplomacy for scientists and diplomats, from junior to senior, rather than relying on external training opportunities in places like the United States or Europe. For science diplomacy to have a lasting impact in the development of the region, in resolving societal, environmental and health issues, it needs to go beyond one-off workshops, seminars, and conferences. Capacity development goes far beyond training^15^: it recognizes the complexity of processes which it aims to influence and the need for multiple knowledges (topical, political, societal, traditional, etc.), provides practical and immersion opportunities to help bridge the gap between theory and practice, and requires a large component of support and follow-up to foster the emergence of vibrant and self-sufficient networks. This can be achieved with the creation of specialized structures within executive and legislative branches, as well as in diplomatic missions, including the deployment of science counselors and attaches to connect the local scientific community with ecosystems of innovation abroad- as well as the diaspora. Science policy fellowship programs, internships, and pairing schemes connecting scientists with legislators and civil servants. Universities to start changing mindsets and cultures that the default career path for a scientist is academia.

One national capacities are established, the next step will be the creation of a regional network of institutions dedicated to building science-policy interfaces, to collectively identify and tackle shared problems in the Latin American region. A pioneer effort in this direction is the Inter-American Institute for Global Change Research (IAI)'s Science, Technology, Policy (STeP) Fellowship Program^16^, an innovative pilot program to enhance human and institutional capacities in IAI member countries, such as Mexico, Argentina, United States, and Canada. The STeP program is training future Latin American and Caribbean leaders to participate in the science-policy interface through hands-on learning supported by professional development and mentorship (the science policy and science diplomacy tracks of the STeP training are delivered by AH and MGS, respectively). Multilateral organizations and regional bodies, such as UNESCO, CELAC, IAI, etc., must coordinate a regional science diplomacy agenda, maximizing connections provided by existing intergovernmental bodies and agreements, and avoiding redundancies and fragmentation. As young scientists are a crucial part of this strategy, early career research networks, associations, and academies, like the Global Young Academy, TWAS Young Affiliates and national academies should also participate in this effort^17^.

We must caution, however, against a too idealized vision of science diplomacy and recognize its limits and even potential negative consequences. Over the last 5 years, nations retreating from multilateralism, trust in science and expertise in decline, and increasing technological competition between major powers has challenged the “romantic view” that has dominated the mainstream discourse on science diplomacy (Rungius and Flink, 2020), prompting intense academic scrutiny of its theoretical and practical frameworks and narratives. Criticism includes neglecting colonialist and imperialist roots of historical scientific cooperation episodes driven by the Global North, such as the Smithsonian Institution, now being reframed as examples of science diplomacy, and discomfort with the idea of science diplomacy because of concerns of compromising academic freedom and scientists being instrumentalized for political purposes (Gual Soler, 2020). As we have seen with the Covid-19 pandemic, science cannot substitute politics, and scientists should not take the role of elected officials. Science and evidence are not the only factors to consider in decision-making, and policymakers must constantly balance competing interests from all sectors of society. It is our responsibility, as science advisors and science diplomats, to provide evidence-informed options to the policy process at the domestic and international levels (Maani and Galea, 2021). To achieve this, we need adequate institutional infrastructures, boundary-spanning professionals and academic incentives to bring science and diplomacy into closer orbits and promote trust-building between their communities, so that they can join forces toward achieving the Sustainable Development Goals for the well-being throughout Latin America and the world.

## Author Contributions

All authors listed have made a substantial, direct and intellectual contribution to the work, and approved it for publication.

## Conflict of Interest

The authors declare that the research was conducted in the absence of any commercial or financial relationships that could be construed as a potential conflict of interest.

## Footnotes

^1^For example, only a handful of countries in Latin American have the expertise, facilities, and resources to routinely sequence circulating SARS-CoV-2 viruses in order to detect emerging variants, which is crucial for a swift and effective public health response. The lack of capacity for genomic surveillance can result in new variants spreading undetected across borders and jeopardize the health security of the entire region.

^2^Science Diplomacy and Leadership Workshop 2017/American Association for the Advancement of Science.

^3^Mason's NCBID and University of Costa Rica Develop Equine Antibody-based Therapeutic to Neutralize Coronavirus/GMU College of Science.

^4^https://www.un.org/Depts/los/clcs_new/submissions_files/submission_criecu_86_2020.htm

^5^http://www.gorgas.gob.pa/historia/

^6^http://www.apanac.org.pa/

^7^https://www.cienciaenpanama.org/

^8^http://www.ccciencias.mx/en

^9^https://www.transdisciplinario.cinvestav.mx/english-profile

^10^Diplomacia científica en América del Norte: México, Estados Unidos y Canadá.

^11^Reunión de Red de Asesoría Científica y Tecnológica para Ministerios de Relaciones Exteriores (FMSTAN).

^12^https://www.aaas.org/news/aaas-and-mexican-scientific-council-sign-collaboration-agreement

^13^https://www.ccciencias.mx/es/asesorias/item/443-mexican-congress.html

^14^https://sectei.cdmx.gob.mx/comunicacion/nota/presentan-programa-de-estancias-de-asesoramiento-gubernamental-pionero-en-america-latina

^15^https://www.diplomacy.edu/blog/what-difference-between-training-and-capacity-development

^16^http://www.iai.int/en/step

^17^https://www.sciencediplomacy.org/editorial/2021/twelve-months-covid-19-shaping-next-era-science-diplomacy
